# Smoking Cigarettes and Tooth Loss in Adults: A Population-Based Analysis

**DOI:** 10.3390/jcm15082903

**Published:** 2026-04-10

**Authors:** Joanna Bagińska, Katarzyna Zienkiewicz, Wojciech Łaguna, Inga Kamińska, Zofia Stachurska, Marlena Dubatówka, Natalia Sieńkowska, Sebastian Sołomacha, Karol Kamiński

**Affiliations:** 1 Department of Dentistry Propaedeutics, Medical University of Bialystok, ul. Szpitalna 30, 15-295 Białystok, Poland; 2 Department of Dentistry Prosthodontics, Medical University of Bialystok, ul. Skłodowskiej 24 a, 15-276 Białystok, Poland; gabieck@o2.pl; 3 Department of Population Medicine and Lifestyle Diseases, Medical University of Białystok, ul. Waszyngtona 15b, 15-269 Białystok, Poland; wojciech.laguna@umb.edu.pl (W.Ł.); zofia.stachurska@umb.edu.pl (Z.S.); marlena.dubatowka@umb.edu.pl (M.D.); natalia.sienkowska@umb.edu.pl (N.S.); sebastian.solomacha@sd.umb.edu.pl (S.S.); karol.kaminski@umb.edu.pl (K.K.); 4 Department of Integrated Dentistry, University of Bialystok, ul. Skłodowskiej 24 a, 15-276 Białystok, Poland; inga.kaminska@umb.edu.pl

**Keywords:** cigarette smoking, tobacco use, tobacco cessation, dentition status, oral health, epidemiology

## Abstract

**Introduction**: Smoking cigarettes is widely recognized as a significant risk factor for tooth loss. **Objectives**: The aim of this study was to assess the association between cigarette smoking, smoking cessation, and the number of remaining teeth in an adult population. **Methods**: Data from the Bialystok Plus population study, collected between November 2018 and January 2024, were analyzed. Participants were stratified into young (20–44 years), middle-aged (45–64 years), and older (65 years and above) groups. The outcome was the number of lost teeth. Smoking status (non-smoker vs. ever smoker and former smoker vs. current smoker) and smoking duration were the main independent variables. Additional variables included age, sex, dental habits and medical history. Risk factor analysis was done with generalized linear models using the negative binomial family. **Results**: Ever smoking was significantly associated with tooth count. Differences in the number of remaining teeth between former and current smokers were observed across age groups but not in the overall population. Current smokers exhibited a significantly greater decline in the number of teeth with an increasing fraction of life spent smoking compared to former smokers. Negative binomial regression models showed that ever smoking, when adjusted for age and diabetes, was a risk factor of tooth loss, but a protective effect of smoking cessation was not confirmed. **Conclusions**: This study confirmed that tobacco addiction is associated with tooth loss. Being a current smoker remained non-significant as a risk factor for tooth loss when compared to former smoker status.

## 1. Introduction

The loss of permanent teeth is considered one of the key indicators of oral health and serves as a proxy for overall health status, as well as a risk marker of mortality [1,2]. The extent of tooth loss depends on the progression of oral diseases and the effectiveness of their management. The number of remaining teeth reflects the availability and quality of dental services in a population. The etiology of tooth loss is multifactorial and includes biological factors such as caries and its complications, periodontal disease, trauma, and oral cancer. Non-biological determinants include causes related to access to dental care, patient preferences, and the method of financing dental treatment. Risk factors of tooth loss include socioeconomic determinants (age, sex, education, standard of living), general health, stimulants, hygiene and dietary habits [3,4,5,6,7,8].

Smoking cigarettes has numerous harmful effects on the oral cavity and is well established as a major risk factor for tooth loss [4,9,10,11,12]. Tobacco use contributes to loss of teeth through several pathways. By reducing the amount of oxygen, it promotes the development of cariogenic and periodontal microbiota [13,14,15]. Although nicotine initially stimulates saliva production, chronic exposure leads to its suppression. A diminished flow rate, along with lower buffering capacity, reduced pH, and elevated concentrations of calcium and phosphorus ions, promotes the accumulation of dental plaque and calculus [14,16]. Smoking modulates the inflammatory mediators and sustains a chronic inflammatory process that leads to the destruction of periodontal ligament and alveolar bone, ultimately resulting in tooth loss [17]. Smokers exhibit higher rates of chronic periodontitis progression and tooth loss, as well as poorer outcomes following both non-surgical and surgical periodontal therapies, compared with non-smokers [18,19,20,21].

The aim of this study was to assess the association between cigarette smoking, smoking cessation, and the number of remaining teeth in the adult population of Bialystok, Poland.

## 2. Materials and Methods

Approval for the study was granted by the Bioethics Committee at the Medical University of Bialystok, No. APK.002.147.2024.

### 2.1. Study Population

We used retrospective data from the Bialystok Plus population study conducted by the Population Research Center of the Medical University of Bialystok, Poland. All patients provided written informed consent prior to enrollment. The STROBE guidelines [22] were applied and presented in Appendix A—Table A1. The cohort was selected to reflect the demographic structure of Bialystok residents aged 20 to 79 (at the time of selection). Data collected between November 2018 and January 2024 were included. The minimum sample size was established at 384 participants based on the following assumptions: the population of Bialystok in 2018—297,459; the proportion of smokers—50%; confidence level—95%; and maximum error—5%. The inclusion criteria comprised availability of data from medical and dental examinations and the availability of data on smoking status, frequency of tooth brushing, and dental visits. The exclusion criterion was the absence of any of the following: dental and medical examinations and questionnaire survey. The study population was stratified into three age groups: young people (from 20 years to 44 years and 11 months), middle-aged people (from 45 years to 64 years and 11 months), and elderly people (65 years and above).

Smoking status was assessed based on participants’ declarations. The population was divided into non-smokers, i.e., those who declared that they had never smoked cigarettes, and ever smokers, i.e., participants who stated that they had smoked cigarettes at some point in their lives. Ever smokers were further categorized as former and current smokers according to their smoking status at the time of participation in the project. For ever smokers who reported the duration of their addiction, the length of smoking in years and the fraction of life spent smoking, expressed as a percentage of years of smoking in relation to age, were determined. Daily exposure to cigarette smoking was assessed on the basis of responses to the following question: “What was/is the highest number of cigarettes you smoke in a day?”

The number of remaining teeth was determined through an oral examination carried out in a dental office under artificial lighting, using a dental mirror and a UNC15 periodontal probe. Third molars were not included in the assessment. A tooth without pathology, a carious or filled tooth, a tooth with a prosthetic crown (single or being part of a bridge), and a dental root were classified as present in the oral cavity. Teeth replaced with an implant, pontics, or removable denture were not considered remaining teeth.

The questionnaire data included the participant’s age, sex, and education; the frequency of tooth brushing; and the time of last dental visit. Diabetes was diagnosed on the basis of medical history and confirmed by medication taken or by the following parameters: fasting blood sugar level of 200 mg/dL or higher, blood sugar level of 200 mg/dL or higher after 2 h in an oral glucose tolerance test (OGTT), or a glycated hemoglobin (HbA1c) value ≥ 6.5. Obesity was diagnosed using the Body Mass Index (BMI) calculated as the ratio of body weight in kilograms to height in meters squared. A BMI ≥ 30 was classified as obesity.

### 2.2. Statistical Analysis

The number of remaining teeth was treated as a count variable. Descriptive tables were reported as median [Q1–Q3]. Pairwise comparisons (never vs. ever; former vs. current) were performed with the two-sided Mann–Whitney U test, and comparisons of more than two groups were performed with the Kruskal–Wallis test. For categorical summaries, group differences were assessed with the χ^2^ test. When expected cell counts were small, the two-sided Fisher’s exact test was used. For covariate-stratified tables, *p*-values were adjusted for multiple testing within each variable using the Holm’s method across the following age groups: total, from 20 years to 44 years and 11 months (20–44), from 45 years to 64 years and 11 months (45–64), and 65 years and more (≥65). Otherwise, unadjusted *p*-values were shown. The risk factor analysis was done with generalized linear models using the negative binomial family (log link, fixed dispersion α = 1.0). For each predictor, two variants were fitted: a crude model (single predictor) and a model adjusted for age and diabetes. Categorical predictors used treatment coding with the advantageous category as a reference. The “current smoker” effect was evaluated only among ever smokers. Coefficients were assessed using Wald tests, and effects were presented as incidence rate ratios (IRRs) with 95% confidence intervals. Two-sided *p* < 0.05 was considered statistically significant. Analyses were conducted in Python 3.12 using SciPy 1.13.1 and statsmodels.

## 3. Results

**Figure 1:**
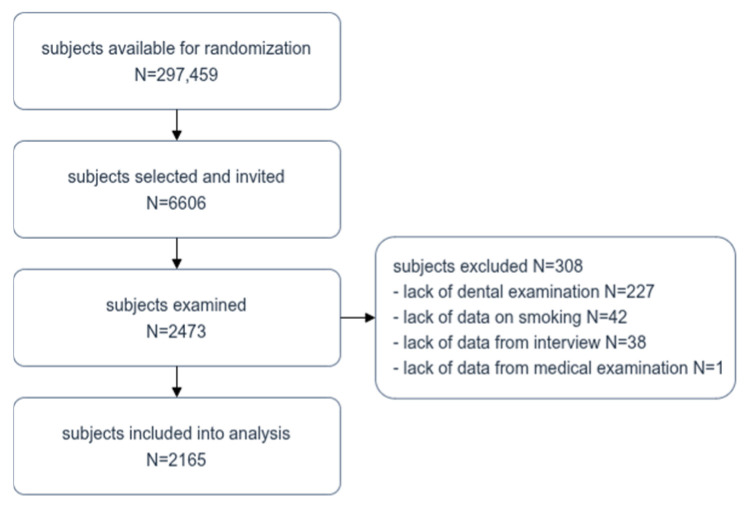
Flow chart of study population selection.

Among the 6606 residents of Bialystok invited to take part in the study, 2473 people participated, giving a response rate of 37.43%. Based on the exclusion criteria, data from 308 individuals were removed from the analysis. The final size of the study population was 2165 individuals. Figure 1 shows a detailed flow chart of the selection of the study population.

The age of the participants ranged from 20 to 82 years (mean 49.2 ± 15.2). The total group included 1213 (56%) women, and 1132 (52.3%) participants had completed higher education. The number of people in each age group was as follows: 914 young people (20–44 years and 11 months old), 809 middle-aged people (45–64 years and 11 months old), and 442 seniors (65 years old and above). The sex structure in the age groups was as follows: 488 (53.4%) women and 426 (46.6%) men in the youngest group, 471 (58.2%) women and 338 (41.8%) men in the middle-aged group, and 254 women (57.5%) vs. 188 men (42.5%) in the senior group. The age groups did not differ statistically based on sex.

The median number of remaining teeth was 25.0 (19.0–28.0). For women, it was 25.0 (19.0–27.0), while men had an average of 25.0 (20.0–28.0) teeth (*p* = 0.11, Mann–Whitney U test). The mean number of teeth in the young population was 28.0 (26.0–28.0); in the middle-aged group, it was 23.0 (19.0–26.0); and it was 14.0 (6.0–20.0) in the elderly group (*p* < 0.001, Kruskal–Wallis test). The mean number of teeth in women in particular age groups was as follows: 28.0 (26.0–28.0), 23.0 (18.0–26.0), 14.0 (6.2–20.0) (*p* < 0.001, Kruskal–Wallis test). The mean number of teeth in men was as follows: 28.0 (26.0–28.0), 24.0 (19.2–26.0) and 14.0 (6.0–20.0) (*p* < 0.001, Kruskal–Wallis test).

In the total cohort, 1227 (56.7%) participants had ever smoked cigarettes. There were 398 (18.4%) current smokers and 829 (38.3%) former smokers. The prevalence of being an ever smoker was 56.8% (519 people) in the youngest group, 55.6% (450 people) in the middle-aged group and 58.4% (258 people) among seniors, and the prevalence of being a current smoker among these age groups was 19.4% (177 people), 18.9% (153 people), and 15.4% (68 people), respectively. In comparisons across age groups, the differences in the proportions of ever versus never smokers, as well as former versus current smokers, were not statistically significant (*p* = 0.6621 and *p* = 0.0653, χ^2^). The mean age of people who had ever smoked was 48.8 ± 15.4 and did not differ significantly from never smokers—49.5 ± 15.1 (*p* = 0.302, Mann–Whitney U test). Current smokers were, on average, 48.0 ± 14.8 years old, whereas former smokers were 50.3 ± 15.2 years old (*p* < 0.05, Mann–Whitney U test). Former smokers reported a smoking duration of 13.9 ± 10.8 years, while current smokers used them for 24.5 ± 14.3 years, and the fraction of life spent smoking was 26.7% ± 17.3 and 47.9% ± 18.6, respectively. Statistically significant differences between former and current smokers were observed across age groups with respect to smoking duration and the fraction of life spent smoking (*p* < 0.001).

Table 1 presents the characteristics of the population according to smoking status. In the total cohort, a statistically significant difference in the count of remaining teeth was observed according to smoking status (never smokers vs. ever smokers), but no such associations were found when comparing former and current smokers. Across all age groups, both ever smokers and current smokers had a significantly lower number of remaining teeth. Sex distribution differed significantly between never and ever smokers (*p* < 0.001), and no differences were identified between former and current smokers. Ever smokers had a lower education level (*p* < 0.001) and had dental visits less frequently (*p* < 0.001) than never smokers. Additionally, ever smokers showed a higher prevalence of obesity and diabetes (*p* = 0.004). Current smokers, compared with former smokers, reported significantly lower educational level (*p* < 0.001) and were more likely to brush teeth irregularly (*p* = 0.043).

Table 2 summarizes data on the teeth count in the whole sample and in the age groups by smoking status. Being an ever smoker had a statistically significant effect on teeth count, and this held at the total sample level regardless of sex, time of last dental visit, diabetes, regular frequency of tooth brushing and lack of obesity. In the youngest group, the lower number of remaining teeth was recorded among male smokers, people who brushed their teeth regularly, those whose last dental visit was over a year ago, participants with a BMI < 30, and individuals without diabetes. In the middle-aged group, ever smokers consistently presented a statistically significant teeth count across all strata. Among seniors, ever smokers, irrespective of sex, and those with secondary or lower education, regular tooth brushing habits, those who visited the dentist over a year ago, and individuals without diabetes and obesity had a lower mean number of teeth.

No significant association between smoking cessation and a higher number of teeth was observed in the overall sample, except among participants without obesity (Table 3). In the youngest age group, a significant association between smoking cessation and greater tooth retention was identified only among individuals with a BMI < 30. Participants aged 45–64 years demonstrated the strongest pattern of associations between the covariates and the number of teeth with regard to quitting smoking vs. continuing tobacco use. Current smokers who were men, less educated, irregularly attended dental visits (>1 year since last visit), had a BMI < 30, and did not have diabetes had fewer remaining teeth than former smokers. Signals by tooth brushing frequency were mixed rather than uniform. In the oldest age group, smoking cessation was associated with a higher number of remaining teeth only among participants without obesity and diabetes.

**Figure 2:**
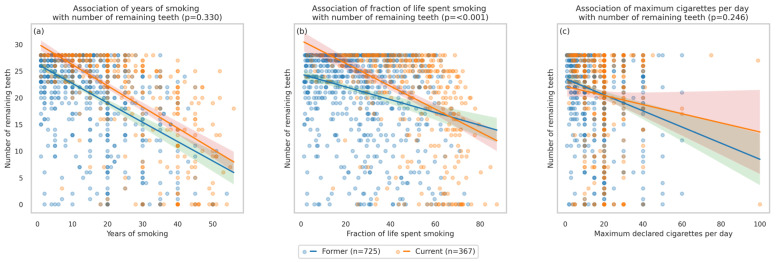
Association between number of remaining teeth and exposure to cigarettes: (**a**) duration of smoking as years of smoking (*p* = 0.330), (**b**) duration of smoking as fraction of life spent smoking (*p* < 0.001), (**c**) maximum cigarettes per day (*p* = 0.246).

Figure 2 presents the association between the number of remaining teeth, the duration of smoking and the maximum daily exposure to cigarettes. Among ever smokers, both exposure metrics of smoking duration showed clear inverse associations with the number of remaining teeth. Years of smoking were negatively related to teeth count in both former and current smokers, but the rate of decline per additional year did not differ among groups (*p* = 0.330). This indicates a broadly similar year-by-year loss pattern in former and current smokers at the sample level. In contrast, the fraction of life spent smoking differed by group. Although the tooth count decreased with increasing fraction in both groups, the decrease was significantly steeper among current smokers (*p* < 0.001). The association of declared maximum cigarettes per day with the tooth count was not significant.

Details of negative binomial regression models identifying risk factors of losing teeth are presented in Table 4. In the total cohort, neither sex nor current smoker status was confirmed as a risk factor of tooth loss. In the model adjusted for age and diabetes, the following factors had a significant impact on the number of remaining teeth: secondary or lower education (β = −0.151, *p* = 0.001), last dental visit more than a year ago (β = −0.144, *p* = 0.003), and ever smoking (β = −0.094, *p* = 0.035). Brushing frequency and obesity were no longer significant, and sex and current smoking remained non-significant among ever smokers. For ever smokers, the basic model indicated age (β = −0.021, *p* < 0.001), secondary or lower education (β = −0.249, *p* < 0.001), last dental visit over one year ago (β = −0.226, *p* < 0.001), obesity ≥ 30 (β = −0.165, *p* = 0.012), diabetes (β = −0.473, *p* < 0.001), and cumulative exposure measures like years of smoking (β = −0.019, *p* < 0.001) and fraction of life spent smoking (β = −0.066, *p* < 0.001) as risk factors. Brushing frequency was not significant, and neither sex nor current smoking reached significance. In the age- and diabetes-adjusted model for ever smokers, significant factors included: secondary or lower education (β = −0.146, *p* = 0.016), last dental visit over one year ago (β = −0.180, *p* = 0.004), years of smoking (β = −0.009, *p* = 0.003), and fraction of life spent smoking (β = −0.004, *p* = 0.017). BMI ≥ 30 and current smoking were not significant after adjustment, and sex remained non-significant.

## 4. Discussion

A key result of the present study is that smoking cigarettes represents an important modifiable factor associated with tooth loss. This finding is in accordance with data from the literature [4,8,9,12,19,23,24,25,26,27,28,29]. Depending on the study, the risk of tooth extraction in smokers was estimated between 1.4 and 2.49 times higher than in non-smokers [4,30,31]. However, not all evidence is consistent, as some studies have reported no statistically significant association between smoking and tooth loss [32,33,34,35,36,37,38,39,40,41,42,43].

According to the literature, smoking cessation improves tooth retention, but a recent meta-analysis indicates that the risk of tooth loss among former smokers remains higher than in never smokers [12,19,24,26,27,29,35,36]. Our results concerning the evidence regarding the impact of smoking cessation on tooth loss were inconclusive. Within each age group, smoking cessation was associated with a reduction in the number of extractions compared to continuing to smoke, and a longer fraction of life spent smoking had a negative impact on the number of teeth when comparing former and current smokers. However, there were no differences in the number of remaining teeth between former and current smokers at the total population level. Moreover, the negative binomial regression models did not identify being a current smoker as a risk factor of tooth loss. This may be explained by the long-term effect of smoking on oral health, even after quitting cigarettes. The period of nicotine abstinence required for the risk of tooth loss to return to that of a non-smoker is 10 to 20 years [24,36,37]. In cross-sectional studies, participants who have only recently quit smoking may influence the reduction in the effect of smoking cessation on the preservation of teeth [28]. The inclusion of such subjects in the analysis may have had an impact on the results. Undoubtedly, the most desirable health behavior is never initiating smoking. Despite the inconsistency of our findings regarding the benefits of smoking cessation for tooth preservation, quitting smoking should be treated as a priority in dental patients [37].

This study provides further support for the multifactorial etiology of tooth loss, with biological factors (age and general diseases), behavioral factors (hygiene habits and dental check-ups), and socio-economic factors (education) playing important roles as risk factors. In our study, negative binomial regression did not identify sex as a risk factor for tooth loss, which is contrary to previous studies [28,35,38,39]. Proper oral hygiene and regular dental care are essential for preventing oral diseases that lead to tooth loss [8,40]. In the SHIP study, last dental visit over 12 months ago, poor general health and poor interdental health were revealed as risk factors of a high number of missing teeth [41]. In elderly Brazilians, last dental visit over 12 months ago and over 24 months ago resulted in prevalence ratios of 2.39 and 2.76 for edentulism [26]. Also, young Finnish adults with improper oral habits were at a greater risk of losing more than six teeth [42]. In our models, a dental visit that took place more than a year ago was a significant predictor of tooth loss, whereas irregular tooth brushing showed no significant correlation. Although previous studies have reported links between BMI and tooth loss, in our cohort, obesity remained insignificant after adjusting for age and diabetes [8,43,44,45].

In negative binomial regression models, age and diabetes were determined as controlling factors. In a crude model, both variables significantly influenced the number of remaining teeth in the overall population and among ever smokers (*p* < 0.001). The chance of preserving teeth decreases with age, and tooth loss peaks in the seventh decade [46,47]. However, a marked decline in extractions is now being observed, particularly among younger adults [26,35,39]. Patients with uncontrolled diabetes are more prone to severe tooth loss and edentulism, but some studies suggest that well-controlled disease may not significantly affect the number of teeth extracted [45,48,49,50,51,52,53]. In the present study, patients were not differentiated according to the type of diabetes (type I and II), the level of disease management, or whether they were newly diagnosed or had been treated for many years. All of the above aspects can affect the severity of tooth loss, which is why diabetes was included as a controlling factor.

A strength of this study is the age-stratified analysis of factors associated with tooth loss. There were only a few variables associated with the number of remaining teeth in ever smokers vs. never smokers in the 20–44 age group. In contrast, a broader range of factors was linked to tooth loss in middle-aged and older individuals with a history of smoking. On the other hand, only a few statistically significant associations were observed for smoking cessation, irrespective of age group.

When comparing young never and ever smokers, a higher number of missing teeth was found among smoking males, those who brushed their teeth regularly, those who visited a dentist irregularly, and those who were not obese and did not have diabetes. In contrast, with the exception of BMI < 30, no associations were found between the variables and the number of preserved teeth among young current and former smokers. In a comparable age group of Japanese individuals, the factors that correlated with tooth loss were being a current smoker regardless of sex, and in women, correlations were found with irregular tooth brushing and being overweight [54]. According to Modin et al. [21], smokers aged ≥25 years with severe periodontal disease had a 3.6-fold higher incidence risk of tooth loss.

Among individuals aged 45–64, ever smokers had fewer teeth compared to never smokers, regardless of other covariates. This finding suggests that in middle-aged adults, who have likely smoked since early adulthood, the detrimental effects of smoking accumulate over time. Similä et al. [37] reported that the majority of middle-aged current smokers had been smoking cigarettes for more than 10 years. They also found that a positive association between time since smoking cessation and tooth count was evident in men but not in women. In the present study, among participants aged 45–64, a comparison between former smokers and current smokers revealed that the only factors significantly associated with tooth loss were a lower level of education and a BMI below 30.

Regarding the oldest age group, no differences in the number of remaining teeth between never and ever smokers were observed among individuals with higher education, those reporting regular dental visits, and those with poor oral hygiene. Hsu at el. [25] found that in elderly subjects, both higher education level and proper oral health behaviors had better general health (i.e., absence of obesity and diabetes), and these factors were associated with a higher number of remaining teeth when comparing ever smokers with never smokers, as well as current smokers with former smokers. These findings may suggest that, in healthy elderly individuals, smoking has a particularly detrimental effect on tooth preservation.

This study has some limitations. First, the analysis was restricted to conventional cigarette smoking, without including alternative nicotine delivery systems such as electronic cigarettes or heated tobacco products. Alternative methods of nicotine delivery have only been available on the market for a few years, and their impact on oral health remains unclear, particularly in comparison with traditional cigarettes [55]. A recent meta-analysis by Thiem et al. [55] suggests that e-cigarettes may be less harmful to periodontal tissues than conventional tobacco products. Since the effect of nicotine on tooth loss is long-term, it is unlikely that the exclusion of alternative methods of nicotine delivery would have a significant impact on the current results. Many studies have shown a dose–response relationship between smoking and tooth loss [19,27,42,56]. The inability to calculate pack-years (resulting from the phrasing of the question on daily cigarette use) should be acknowledged as a limitation of the present study. Instead, the severity of tobacco exposure was estimated on the basis of smoking duration in years and the fraction of life spent smoking, and it was found to be a significant risk factor for tooth loss.

## 5. Conclusions

The results of this study show an association between tobacco addiction and the ability to maintain dentition. Being an ever smoker increased the risk of tooth loss regardless of age and diabetes, but being a current smoker remained non-significant as a risk factor for tooth loss in comparison to former smokers. This study highlights the important role that dentists can play in tobacco prevention by informing patients about the greater risk of tooth loss due to cigarette smoking.

## Figures and Tables

**Table 1:** Characteristics of the population according to smoking status (^a^ test U Mann–Whitney; ^b^ χ^2^) (median (IQR 25–75), N (%)).

		Never Smoker N = 938	Ever Smoker N = 1227	*p*	Former Smoker N = 829	Current Smoker N = 398	*p*
number of teeth	total	26.0 (21.0–28.0)	24.0 (18.0–27.0)	<0.001 ^a^	24.0 (18.0–27.0)	24.0 (16.0–27.0)	0.136 ^a^
	20–44	28.0 (26.0–28.0)	28.0 (26.0–28.0)	<0.010 ^a^	28.0 (26.0–28.0)	27.0 (25.0–28.0)	0.027 ^a^
	45–64	25.0 (21.0–27.0)	22.0 (17.0–25.0)	<0.001 ^a^	23.0 (18.0–25.0)	21.0 (13.0–25.0)	0.002 ^a^
	over 64	15.5 (8.8–21.0)	13.0 (5.0–19.8)	0.001 ^a^	13.5 (6.0–20.0)	9.0 (2.8–18.0)	0.035 ^a^
sex	women	594 (63.3)	619 (50.4)	<0.001 ^b^	427 (51.5)	192 (48.2)	0.300 ^b^
	men	344 (36.7)	608 (49.6)		402 (48.5)	206 (51.8)	
education	secondary or lower	372 (39.7)	661 (53.9)	<0.001 ^b^	413 (49.8)	248 (62.3)	<0.001 ^b^
	university	566 (60.3)	566 (46.1)		416 (50.2)	150 (37.7)	
frequency of tooth brushing	once a day or less	155 (16.5)	233 (19.0)	0.142 ^b^	144 (17.4)	89 (22.4)	0.043 ^b^
	two times a day or more	783 (83.5)	994 (81.0)		685 (82.6)	309 (77.6)	
last dental visit	over one year	242 (25.8)	411 (33.5)	<0.001 ^b^	263 (31.7)	148 (37.2)	0.061 ^b^
	within last year	696 (74.2)	816 (66.5)		566 (68.3)	250 (62.8)	
BMI	≥30	207 (22.1)	338 (27.5)	0.004 ^b^	238 (28.7)	100 (25.1)	0.195 ^b^
	<30	731 (77.9)	889 (72.5)		591 (71.3)	298 (74.9)	
diabetes	yes	82 (8.7)	155 (12.6)	0.004 ^b^	108 (13.0)	47 (11.8)	0.583 ^b^
	no	856 (91.3)	1072 (87.4)		721 (87.0)	351 (88.2)	

**Table 2:** Number of remaining teeth according to smoking status (never smokers vs. current smokers).

Variable		Age Group	Never Smokers Median (25–75)	Ever Smokers Median (25–75)	*p*_adj
sex	women	total	25.0 (20.0–28.0)	24.0 (17.0–27.0)	<0.001
		20–44	28.0 (26.0–28.0)	27.0 (26.0–28.0)	0.118
		45–64	24.0 (21.0–27.0)	22.0 (16.0–25.0)	<0.001
		over 64	14.5 (9.0–21.0)	13.0 (4.8–20.0)	0.043
	men	total	26.0 (22.0–28.0)	24.0 (18.0–28.0)	<0.001
		20–44	28.0 (26.0–28.0)	28.0 (26.0–28.0)	0.017
		45–64	25.0 (22.0–27.0)	23.0 (18.0–25.0)	<0.001
		over 64	18.0 (8.2–21.0)	12 (6.0–19.0)	0.017
education	secondary or lower	total	22.0 (15.0–26.0)	21.0 (13.0–26.0)	0.219
		20–44	28.0 (25.0–28.0)	27.0 (24.0–28.0)	0.079
		45–64	22.0 (18.0–25.0)	21.0 (14.0–24.0)	0.011
		over 64	14.0 (7.0–20.0)	11.0 (4.0–17.0)	0.004
	university	total	27.0 (24.0–28.0)	26.0 (23.0–28.0)	0.006
		20–44	28.0 (27.0–28.0)	28.0 (27.0–28.0)	0.496
		45–64	26.0 (24.0–27.0)	24.0 (21.0–26.0)	<0.001
		over 64	20.0 (11.5–22.0)	17.0 (9.0–22.0)	0.386
frequency of tooth brushing	once a day or lower	total	24.0 (17.5–27.0)	22.0 (11.0–26.0)	0.082
		20–44	28.0 (26.0–28.0)	28.0 (25.0–28.0)	0.591
		45–64	24.0 (20.0–26.0)	21.0 (15.0–25.0)	0.035
		over 64	14.0 (5.0–20.0)	11.0 (4.2–17.0)	0.153
	two times a day or more	total	26.0 (22.0–28.0)	24.0 (19.0–28.0)	<0.001
		20–44	28.0 (26.0–28.0)	28.0 (26.0–28.0)	0.011
		45–64	25.0 (21.0–27.0)	23.0 (17.8–25.0)	<0.001
		over 64	16.0 (10.0–21.0)	14.0 (6.0–20.0)	0.012
last dental visit	over one year	total	25.0 (19.0–28.0)	22.0 (10.5–26.0)	<0.001
		20–44	28.0 (27.0–28.0)	27.0 (25.0–28.0)	0.003
		45–64	24.0 (19.0–27.0)	21.0 (13.5–24.0)	<0.001
		over 64	13.5 (6.0–20.0)	8.0 (1.0–17.0)	0.003
	during last year	total	26.0 (22.0–28.0)	25.0 (20.0–28.0)	0.037
		20–44	28.0 (26.0–28.0)	28.0 (26.0–28.0)	0.349
		45–64	25.0 (22.0–27.0)	23.0 (18.0–25.0)	<0.001
		over 64	16.5 (10.8–21.0)	16.0 (9.0–21.0)	0.477
obesity	≥30	total	22.0 (17.0–26.0)	21.0 (13.2–25.8)	0.279
		20–44	27.0 (25.0–28.0)	27.0 (25.0–28.0)	0.915
		45–64	23.0 (20.8–26.0)	21.0 (15.2–24.0)	<0.001
		over 64	14.0 (7.0–20.0)	11.0 (5.0–19.0)	0.188
	<30	total	26.0 (23.0–28.0)	25.0 (20.0–28.0)	<0.001
		20–44	28.0 (26.2–28.0)	28.0 (26.0–28.0)	0.011
		45–64	25.0 (22.0–27.0)	23.0 (18.0–25.2)	<0.001
		over 64	17.5 (10.2–21.0)	13.0 (5.2–20.0)	0.011
diabetes	yes	total	19.0 (9.0–23.0)	16.5 (6.0–22.0)	0.004
		20–44	25.0 (23.5–26.5)	27.5 (26.2–28.0)	>0.999
		45–64	23.0 (17.8–26.2)	18.0 (9.0–23.5)	0.001
		over 64	12.5 (6.2–20.0)	9.0 (5.0–17.0)	0.124
	no	total	26.0 (22.0–28.0)	25.0 (19.0–28.0)	<0.001
		20–44	28.0 (26.0–28.0)	28.0 (26.0–28.0)	0.011
		45–64	25.0 (21.0–27.0)	23.0 (18.0–25.0)	<0.001
		over 64	16.0 (9.0–21.0)	13.0 (6.0–20.0)	0.011

**Table 3:** Number of remaining teeth according to smoking status (former smokers vs. current smokers).

Variable		Age Group	Former Smokers Median (25–75)	Current Smokers Median (25–75)	*p*_adj
sex	women	total	24.0 (18.0–27.0)	23.0 (13.0–27.0)	0.138
		20–44	28.0 (26.0–28.0)	27.0 (25.0–28.0)	0.138
		45–64	22.0 (18.0–25.0)	20.5 (12.2–25.0)	0.138
		over 64	15.0 (6.0–20.0)	7.0 (2.0–16.0)	0.091
	men	total	24.0 (18.0–28.0)	24.0 (18.0–27.8)	>0.999
		20–44	28.0 (26.0–28.0)	27.0 (25.0–28.0)	0.404
		45–64	23.0 (20.0–26.0)	21.0 (15.5–25.0)	0.030
		over 64	12.0 (6.0–19.0)	13.0 (6.5–18.0)	>0.999
education	secondary or lower	total	21.0 (13.0–25.0)	22.0 (13.0–26.0)	0.760
		20–44	27.0 (24.5–28.0)	27.0 (24.0–28.0)	0.760
		45–64	21.5 (16.2–24.0)	18.0 (9.0–23.0)	<0.001
		over 64	11.0 (5.0–18.0)	7.0 (1.0–14.5)	0.153
	university	total	27.0 (23.0–28.0)	26.0 (22.0–28.0)	>0.999
		20–44	28.0 (27.0–28.0)	28.0 (26.5–28.0)	>0.999
		45–64	24.0 (21.5–26.0)	25.0 (21.0–26.0)	>0.999
		over 64	18.0 (10.5–22.0)	13.0 (7.0–21.0)	>0.999
frequency of tooth brushing	once a day or lower	total	21.0 (11.0–27.0)	22.0 (14.0–26.0)	0.879
		20–44	28.0 (26.0–28.0)	27.0 (24.0–28.0)	0.320
		45–64	22.0 (18.0–26.0)	18.0 (14.0–24.0)	0.083
		over 64	11.0 (5.0–17.0)	7.0 (3.0–15.5)	0.577
	two times a day or more	total	24.0 (20.0–28.0)	25.0 (17.0–27.0)	0.205
		20–44	28.0 (26.0–28.0)	27.0 (25.0–28.0)	0.191
		45–64	23.0 (18.0–25.0)	21.0 (13.0–25.0)	0.086
		over 64	15.5 (6.0–21.0)	9.5 (2.5–19.0)	0.150
last dental visit	over one year	total	23.0 (12.0–27.0)	20.0 (7.0–26.0)	0.108
		20–44	27.0 (25.0–28.0)	27.0 (24.0–28.0)	0.194
		45–64	23.0 (18.0–25.0)	17.0 (5.0–22.2)	0.001
		over 64	10.5 (2.0–17.0)	7.0 (0.0–12.0)	0.194
	during last year	total	25.0 (20.0–28.0)	25.0 (20.0–28.0)	>0.999
		20–44	28.0 (26.0–28.0)	27.0 (26.0–28.0)	0.407
		45–64	23.0 (19.0–25.0)	22.0 (17.0–25.0)	>0.999
		over 64	16.0 (9.8–21.0)	14.0 (8.0–20.0)	>0.999
obesity	≥30	total	25.0 (21.0–28.0)	21.0 (15.5–26.2)	>0.999
		20–44	21.0 (13.0–25.0)	27.5 (25.8–28.0)	>0.999
		45–64	27.0 (25.0–28.0)	19.5 (16.0–23.0)	0.477
		over 64	21.0 (15.0–24.0)	10.0 (6.2–18.8)	>0.999
	<30	total	25.0 (21.0–28.0)	25.0 (17.0–27.0)	0.017
		20–44	28.0 (26.0–28.0)	27.0 (25.0–28.0)	0.013
		45–64	24.0 (20.0–26.0)	22.0 (13.0–25.0)	0.009
		over 64	15.0 (6.0–21.0)	8.5 (0.5–16.8)	0.017
diabetes	yes	total	16.5 (6.0–22.0)	15.5 (5.0–20.8)	>0.999
		20–44	28.0 (27.5–28.0)	24.0 (22.5–25.5)	0.492
		45–64	19.0 (10.5–24.0)	18.0 (8.8–19.5)	>0.999
		over 64	11.0 (6.0–17.0)	5.5 (1.5–11.8)	>0.999
	no	total	25.0 (20.0–28.0)	25.0 (17.8–27.0)	0.114
		20–44	28.0 (26.0–28.0)	27.0 (25.0–28.0)	0.114
		45–64	23.0 (19.0–25.0)	22.0 (14.0–25.0)	0.033
		over 64	15.0 (6.0–20.0)	10.5 (3.0–18.0)	0.045

**Table 4:** Risk factor analysis for total population and in ever smokers. Negative binomial regression (crude model and adjusted by age and diabetes).

Risk Factor	β	IRR	95% CI	*p*	β	IRR_.adj_	95% CI	*p*
	total							
age	−0.018	0.982	0.980–0.985	<0.001	_	_	_	_
sex male	0.017	0.983	0.902–1.072	0.704	−0.006	0.994	0.911–1.084	0.889
education secondary or lower	−0.259	0.772	0.708–0.841	<0.001	−0.151	0.860	0.786–0.941	0.001
frequency of tooth brushing once a day or lower	−0.136	0.873	0.780–0.977	0.018	−0.078	0.925	0.826–1.036	0.179
last dental visit over one year	−0.167	0.846	0.770–0.930	<0.001	−0.144	0.866	0.788–0.952	0.003
BMI ≥30	−0.177	0.837	0.758–0.925	<0.001	−0.027	0.974	0.878–0.08	0.615
diabetes yes	−0.444	0.642	0.539–0.764	<0.001	_	_	_	_
ever smoker yes	−0.087	0.915	0.839–0.998	0.045	−0.094	0.911	0.835–0.994	0.035
current smoker yes	−0.052	0.950	0.840–1.073	0.408	−0.113	0.893	0.79–1.01	0.072
	ever smokers							
age	−0.021	0.976	0.976–0.983	<0.001	_	_	_	_
sex male	0.021	1.021	0.91–1.1145	0.726	0.011	1.011	0.901–1.343	0.850
education secondary or lower	−0.249	0.779	0.695–0.875	<0.001	−0.146	0.865	0.768–0.973	0.016
frequency of tooth brushing once a day or lower	−0.144	0.866	0.748–1.003	0.054	−0.095	0.91	0.785–1.054	0.208
last dental visit over one year	−0.226	0.798	0.706–0.901	<0.001	−0.180	0.835	0.738–0.945	0.004
BMI ≥30	−0.165	0.848	0.745–0.964	0.012	−0.03	0.971	0.85–1.108	0.660
diabetes yes	−0.473	0.623	0.502–0.773	<0.001	_	_	_	_
current smoker yes	−0.052	0.950	0.84–1.073	0.408	−0.113	0.893	0.79–1.01	0.072
years of smoking	−0.019	0.981	0.977–0.986	<0.001	−0.009	0.992	0.986–0.997	0.003
fraction of life spent smoking	−0.066	0.993	0.990–0.996	<0.001	−0.004	0.996	0.993–0.999	0.017

Note: – the variable included in the model as an adjustment covariate (the coefficient is not reported).

## Data Availability

The original contributions presented in this study are included in the article. Further inquiries can be directed to the corresponding author.

## Abbreviations

The following abbreviations are used in this manuscript:

OGTT	oral glucose tolerance test
HbA1c	hemoglobin A1C
BMI	body mass index
IRR	incidence rate ratios

## Appendix A

**Table A1:** STROBE checklist.

	Item No	Recommendation	Page
Title and abstract	1	(a) Indicate the study’s design with a commonly used term in the title or the abstract	1–2
		(b) Provide in the abstract an informative and balanced summary of what was done and what was found	1–2
Introduction			
Background/rationale	2	Explain the scientific background and rationale for the investigation being reported	2
Objectives	3	State specific objectives, including any prespecified hypotheses	2
Methods			
Study design	4	Present key elements of study design early in the paper	2–3
Setting	5	Describe the setting, locations, and relevant dates, including periods of recruitment, exposure, follow-up, and data collection	3
Participants	6	(a) Give the eligibility criteria, and the sources and methods of selection of participants	3
Variables	7	Clearly define all outcomes, exposures, predictors, potential confounders, and effect modifiers. Give diagnostic criteria, if applicable	3
Data sources/measurement	8	For each variable of interest, give sources of data and details of methods of assessment (measurement). Describe comparability of assessment methods if there is more than one group	3
Bias	9	Describe any efforts to address potential sources of bias	3
Study size	10	Explain how the study size was arrived at	3
Quantitative variables	11	Explain how quantitative variables were handled in the analyses. If applicable, describe which groupings were chosen and why	3
Statistical methods	12	(a) Describe all statistical methods, including those used to control for confounding	3–4
		(b) Describe any methods used to examine subgroups and interactions	3–4
		(c) Explain how missing data were addressed	NA
		(d) If applicable, describe analytical methods taking account of sampling strategy	3
Results			
Participants	13	(a) Report numbers of individuals at each stage of study—e.g., numbers potentially eligible, examined for eligibility, confirmed eligible, included in the study, completing follow-up, and analysed	Figure 1
		(b) Give reasons for non-participation at each stage	Figure 1
		(c) Consider use of a flow diagram	Figure 1
Descriptive data	14	(a) Give characteristics of study participants (e.g., demographic, clinical, social) and information on exposures and potential confounders	4
		(b) Indicate number of participants with missing data for each variable of interest	Figure 1
Outcome data	15	Report numbers of outcome events or summary measures	
Main results	16	(a) Give unadjusted estimates and, if applicable, confounder-adjusted estimates and their precision (e.g., 95% confidence interval). Make clear which confounders were adjusted for and why they were included	4–11
		(b) Report category boundaries when continuous variables were categorized	NA
		(c) If relevant, consider translating estimates of relative risk into absolute risk for a meaningful time period	NA
Other analyses	17	Report other analyses done—e.g., analyses of subgroups and interactions, and sensitivity analyses	NA
Discussion			
Key results	18	Summarise key results with reference to study objectives	11–14
Limitations	19	Discuss limitations of the study, taking into account sources of potential bias or imprecision. Discuss both direction and magnitude of any potential bias	13–14
Interpretation	20	Give a cautious overall interpretation of results considering objectives, limitations, multiplicity of analyses, results from similar studies, and other relevant evidence	12–13
Generalisability	21	Discuss the generalisability (external validity) of the study results	13
Other information			
Funding	22	Give the source of funding and the role of the funders for the present study and, if applicable, for the original study on which the present article is based	14
